# The Relationship between Perioperative Blood Transfusion and Postoperative Delirium in Patients Undergoing Spinal Fusion Surgery: Clinical Data Warehouse Analysis

**DOI:** 10.3390/medicina58020268

**Published:** 2022-02-11

**Authors:** Young-Suk Kwon, Jong-Ho Kim, Jae-Jun Lee, Eun-Min Seo

**Affiliations:** 1 Division of Big Data and Artificial Intelligence, Institute of New Frontier Research, Chuncheon Sacred Heart Hospital, Hallym University College of Medicine, Chuncheon 24253, Korea; gettys@hallym.or.kr (Y.-S.K.); iloveu59@hallym.or.kr (J.-J.L.); 2 Department of Anesthesiology and Pain Medicine, College of Medicine, Chuncheon Sacred Heart Hospital, Hallym University College of Medicine, Chuncheon 24253, Korea; poik99@hallym.or.kr; 3 Department of Anesthesiology and Pain Medicine, College of Medicine, Kangwon National University, Chuncheon 24253, Korea; 4 Department of Orthopedic Surgery, Chuncheon Sacred Heart Hospital, Hallym University College of Medicine, Chuncheon 24253, Korea

**Keywords:** postoperative delirium, spinal fusion surgery, clinical data warehouse, risk factor, blood transfusion

## Abstract

*Background and Objectives:* For preventing postoperative delirium (POD), identifying the risk factors is important. However, the relationship between blood transfusion and POD is still controversial. The aim of this study was to identify the risk factors of POD, to evaluate the impact of blood transfusion in developing POD among people undergoing spinal fusion surgery, and to show the effectiveness of big data analytics using a clinical data warehouse (CDW). *Materials and Methods:* The medical data of patients who underwent spinal fusion surgery were obtained from the CDW of the five hospitals of Hallym University Medical Center. Clinical features, laboratory findings, perioperative variables, and medication history were compared between patients without POD and with POD. *Results:* 234 of 3967 patients (5.9%) developed POD. In multivariate logistic regression analysis, the risk factors of POD were as follows: Parkinson’s disease (OR 5.54, 95% CI 2.15–14.27; *p* < 0.001), intensive care unit (OR 3.45 95% CI 2.42–4.91; *p* < 0.001), anti-psychotics drug (OR 3.35 95% CI 1.91–5.89; *p* < 0.001), old age (≥70 years) (OR 3.08, 95% CI 2.14–4.43; *p* < 0.001), depression (OR 2.8 95% CI 1.27–6.2; *p* < 0.001). The intraoperative transfusion (OR 1.1, 95% CI 0.91–1.34; *p* = 0.582), and the postoperative transfusion (OR 0.91, 95% CI 0.74–1.12; *p* = 0.379) had no statistically significant effect on the incidence of POD. *Conclusions:* There was no relationship between perioperative blood transfusion and the incidence of POD in spinal fusion surgery. Big data analytics using a CDW could be helpful for the comprehensive understanding of the risk factors of POD, and for preventing POD in spinal fusion surgery.

## 1. Introduction

Postoperative delirium (POD) is a mental disorder characterized by acute and fluctuating course of consciousness disturbance [1,2]. It is usually occurs within 5 days after surgery, especially in the first 24 to 48 h. POD may lead to higher morbidity and mortality, higher hospitalization, and higher healthcare costs [3,4,5,6,7]. Therefore, preventing POD is important for improving a patient’s prognosis.

Although the risk factors for POD are well-known, many studies about the risk factors for POD are still being reported. It might result in an increase in the numbers of risk factors and variety of risk factors among studies and type of surgery [1,3]. In previous studies, the preoperative anemia or blood transfusion associated with intraoperative blood loss may predict the development of POD [5,8,9,10]. However, other studies have reported inconsistent findings (There was no significant relationship between preoperative anemia and blood transfusion and POD) [11,12]. The relationship between blood transfusion and POD is still controversial in patients undergoing specified surgery.

Too many risk factors can make it difficult to comprehensively evaluate all possible factors (i.e., epidemiologic, laboratory, perioperative factors, and medication use, etc.). It is a time-consuming task to assess the level of each risk factor properly and to identify risk factors. Recently, medical records have rapidly become digitized, and big data analytics has been applied in the healthcare practices and research. It provides a tool to analyze structured and unstructured data quickly and precisely [13].

Therefore, the aim of this study was to identify the risk factors of POD, to evaluate the impact of blood transfusions in developing POD among people undergoing spinal fusion surgery, and to show the effectiveness of big data analytics using a clinical data warehouse (CDW).

## 2. Materials and Methods

### 2.1. Ethics, Study Design and Setting

Ethical approval for this study (2021-09-005) was provided by the Clinical Research Ethics Committee of Chuncheon Sacred Heart Hospital, Hallym University, Chuncheon and Republic of Korea. Although it included vulnerable participants, because it was a historical cohort analysis of clinical data acquired during treatment processes that had already been completed, informed consent was waived for all subjects. All data were obtained from the clinical data warehouse (CDW) of the five hospitals of Hallym University Medical Center. The CDW is a database of medical records, prescriptions, and test results from Hallym University Medical Center, spanning outpatient and inpatient data. Patients can be searched according to prescriptions, examinations, and diagnosis, among other variables. The CDW provides medical records in an unstructured format in addition to the patient’s test, transfusion, and drug administration records.

### 2.2. Participants

The study subjects were patients 18 years of age or older who underwent spinal fusion surgery under general anesthesia during 11 years (1 January 2011–31 July 2021) were eligible for analysis. Exclusion criteria are as follows: patients taking anti-delirium drugs prior to surgery, patients with delirium before surgery, patient unconscious after surgery, patients who underwent other surgeries at the same time, patients receiving sedation after surgery, and patients with missing medical records.

### 2.3. Exposure Variables and Primary Outcomes

The exposure variable was intraoperative transfusion amount of red blood cells and transfusion of red blood cells during 48 h after surgery. Pack was used as the unit of blood transfusion. The primary outcome was POD during 48 h after surgery. POD was assessed by using the short-form Korean Nursing Delirium Screening Scale on a daily basis by nurse [14]. Once patients were suspected to be POD by a nurse, the patients received psychiatric counseling and the diagnosis of POD was confirmed. POD patients were defined as patients with words indicating predetermined specific symptoms and signs of delirium in the consultation notes among all individuals who received postoperative psychiatric counseling [13]. We have created a list of words for specific symptoms and symbols (Appendix A). Therefore, our smart CDW defined patients with these words in electronic medical records (EMR). That way, other psychiatric patients with similar symptoms to delirium could be excluded as much as possible.

We additionally calculated odds-ratios of perioperative risk factors including intra- and postoperative red blood cell transfusion for patients.

### 2.4. Other Variables

Other perioperative covariates were used to adjust for confounding and bias in intra and postoperative red blood cell transfusion. The covariates included patient’s general characteristics, preoperative medication, morbidities and laboratory test, characteristics of anesthetics and surgery, and postoperative laboratory test and transfusion except red blood cells.

#### 2.4.1. Preoperative Factors

Preoperative risk factors were: old age (≥70 years), male, body mass index, American society of Anesthesiology physical status > 2, emergency surgery, preoperative morbidities(hypertension, diabetes, heart disease, stroke, cancer, Parkinson disease, dementia, depression, kidney disease, liver disease, insomnia, sleep disorder), medication (calcium channel blocker, beta blocker, angiotensin converting enzyme inhibitors, angiotensin-receptor blockers, anti-depressants, hypnotics, anti-psychotics, non-steroidal anti-inflammatory drug, analgesic except non-steroidal anti-inflammatory drug, muscle relaxants, steroid, anti-platelet, anti-coagulants, anti-hyperlipidemic, anti-Parkinson, anti-histamine, anti-vertigo, genitourinary drug, h2-blocker, miscellaneous), alcohol, smoking, laboratory test (hemoglobin, aspartate aminotransferase, alanine aminotransferase, sodium, potassium, uric acid, blood urea nitrogen, creatinine, albumin). We have created a list of drugs in Appendix B.

#### 2.4.2. Intraoperative Factors

Intraoperative risk factors were: maintenance anesthetics, opioid, midazolam, oliguria (<0.5 mL/kg/h), fluid balance, estimated blood loss, transfusion amount of fresh frozen plasma and platelet, surgical site (cervix, thorax and lumbar), surgical range (fusion level), surgical time and transfusion amount of fresh frozen plasma and platelet concentration.

#### 2.4.3. Postoperative Factors

Postoperative risk factors were: intensive care unit admission, patient-controlled analgesia, transfusion amount of fresh frozen plasma and platelet, laboratory test (hemoglobin, aspartate aminotransferase, alanine aminotransferase, sodium, potassium, uric acid, blood urea nitrogen, creatinine, albumin, erythrocyte sedimentation rate, C-reactive protein) and maximum body temperature.

When two surgical sites were included, the lower part was marked as the surgical site.

### 2.5. Statistics

Statistical significance was evaluated by independent *t*-test or Mann–Whitney test, and Chi-square test. Continuous data were presented as median and interquartile ranges (IQRs), while categorical data were presented as frequencies and percentages. Continuous variables were analyzed by independent *t*-test or Mann–Whitney test, and chi-square analysis was used for categorical data. We calculated the unadjusted and adjusted odds ratio and 95% confidence intervals for developing POD using Multivariate logistic regression with backward elimination. Multivariate logistic regression with backward elimination was performed on variables that showed *p* < 0.20 in univariate logistic regression. We decided on *p* = 0.20 as the threshold in multivariate analysis. All *p*-values were two-sided, and a *p*-value < 0.05 was considered indicative of statistical significance. IBM SPSS Statistics (version 26.0; IBM Corp., Armonk, NY, USA) was used for the statistical analyses.

## 3. Results

**Figure 1:**
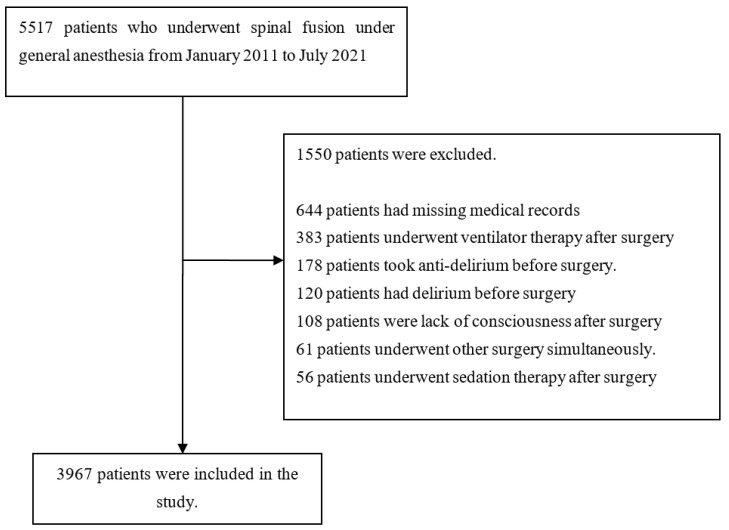
Flow chart.

From January 2011 to July 2021, 5517 patients underwent spinal fusion surgery under general anesthesia at one of the five hospitals of Hallym University. In total, 1550 patients met at least one exclusion criterion. Thus, 3967 patients were initially included in the study. The flow chart is summarized in Figure 1.

After surgery 234 (5.9%) patients developed POD. Preoperative, intraoperative and postoperative risk factors of patients are summarized in Table 1.

Of the patients who developed POD, 119 (50.9%) received intraoperative red blood cell transfusions. Of the patients without POD, 1032 (27.6%) received intraoperative blood transfusions. Of the patients who developed POD, 42 (17.9%) received postoperative red blood cell transfusions. Of the patients without POD, 431 (11.5%) received postoperative blood transfusions. Red blood cell transfusion distributions are summarized in Table 2.

The intraoperative red blood cell transfusion volume (Odds ratio (95% confidence interval), 1.1 (0.91–1.34)), and the postoperative red blood cell transfusion volume (Odds ratio (95% confidence interval), 0.91 (0.74–1.12)) had no statistically significant effect on the incidence of POD. In sensitivity analysis, intraoperative red blood cell transfusion and postoperative red blood cell transfusion did not have a statistically significant effect on the incidence of POD. The odds ratio for developing POD is summarized in Table 3.

The odds ratios of other factors for developing POD are summarized in Table 4.

## 4. Discussion

POD is a serious condition after surgery in patients. Patients who develop POD have a higher risk of severe complications, such as a fall, myocardial infarction, pulmonary edema, pneumonia, and pressure ulcers [15]. This can result in longer hospital stay, higher health-care cost, and functional decline [16,17]. Therefore, preventing POD is important for improving a patient’s prognosis, and recognizing the risk factors of POD is essential to prevent it [3,4,5,6,7]. The cause of POD is associated with multiple risk factors, such as preexisting dementia, functional impairment, preoperative anemia, drug or alcohol abuse, intraoperative blood loss, surgical duration > 3 h, general anesthesia, electrolyte imbalance, low albumin, postoperative sleep disorders, and frequent hypotension [5,18,19,20].

However, questions remain about the generalizability and application of risk factors of POD to patients undergoing specified surgery, because risk factors of POD were determined on the basis of the analysis of various types of surgeries. According to studies, the well-known risk factors of POD were sometimes denied [10,11,12]. Consequently, the incidences of POD were various among studies [5,10,11,12].

The incidence of POD varied between 9% and 41% depending on the type of surgery [1,2,5,10,11,12]. In this study, the incidence of POD was 5.9% and lower than other studies [5,18,19,20]. Fineberg et al. reported the incidence rate of 11.8% in 570,000 patients who underwent lumbar decompression surgery or lumbar fusion [5]. These differences in POD incidence seem dependent on the diagnostic methods used. In this study, patients were diagnosed via psychiatric consultations when symptoms developed, some patients with milder symptoms may have been missed during this process. This has an advantage in the analysis based on a solid diagnosis.

We identified the risk factors of POD in spinal fusion surgery through a comprehensive evaluation using big data. Old age (≥70 years), Parkinson’s disease, depression, intensive care unit stay, and anti-psychotics drug were significantly associated with POD (Table 4). Old age, especially in patients over 65, is the most frequently identified risk factor for POD [18,21]. The higher incidence of POD in the advanced age is associated with increased comorbidities as well as age-related physical and neurobiological changes (permanent damage of the nervous system due to aging) [3].

Some studies have been reported that lower body mass index (BMI) was a significant risk factor of POD, and it is associated with an under-nutrition status [22]. Also, negative findings have been reported [23,24,25]. In this study, there was no significant difference in mean BMI scores between patients with POD and those without POD. Because patients with lower or higher body mass index tend to have many comorbidity. Patients with lower or higher body mass index are frequently excluded in more aggressive surgery like spinal fusion surgery. Consequentially, there was no relationship between BMI and POD in this study. Previous history of Parkinson’s disease is a well-known risk factor of POD in various kinds of surgery [3,21,24]. Parkinson’s disease also was a significant risk factor of POD in this study.

Although, decreased brain function and alpha synucleinopathy were thought to possible mechanisms, the precise role of Parkinson’s disease is poorly understood [21,26,27].

Generally, polypharmacy, medications with anticholinergic activity, and anti-psychotics medications are related to POD [28,29]. We investigated drugs that had been administered before surgery. Our results were no different from those of previous studies [28,29,30].

The relationship between emergency surgery and POD is still controversial. Some studies suggested that emergency surgery is related to POD [31]. That is because, emergency surgery could not give environmental conditions that made patients feel comfortable. However, Malik et al., who assessed the risk factors for delirium in cases of femoral fractures, reported higher POD incidence in non-emergency surgeries [32]. In this study, there was no significant relationship between emergency surgery and POD. Usually, emergency spine surgery was performed in cases of traumatic spine fracture, incomplete spinal cord injury, and cauda equina syndrome. These cases were accompanied by severe pain. The reason may be associated with early pain control through emergency surgery, because pain is a well-known risk factor of POD [13].

Anemia (defined as hemoglobin level less than 12 g/dL in women and less than 13 g/dL in men by the World Health Organization) was reported to be a risk factor of POD [18,33,34].

Intra- and postoperative anemia is related to intraoperative blood loss. If the large blood loss during surgery leads to unstable vital signs in patients a blood transfusion is carried out. Therefore, intraoperative blood loss is highly correlated to blood transfusions. The POD-related intraoperative blood loss may be associated with decreased cerebral blood flow, impaired metabolism, and inflammatory reactions [35]. The lack of oxygen delivery caused by the decreased cerebral blood flow may cause POD [2]. Also, a blood transfusion itself may cause POD, because dysregulation of cytokines and activated systemic inflammation due to blood transfusion is associated to POD [36,37].

However, the relationship between blood transfusion and POD is still controversial [38,39,40,41].

Some studies reported that lower hemoglobin (<6.0 g/dL) was a risk factor for POD in older patients undergoing hip-fracture operation and blood transfusion may reduce the incidence of delirium [38,39]. However, the findings were not supported by other studies [40,41].

Hemoglobin levels in elective surgery like spinal fusion surgery are usually much higher than those in acute conditions (hip fractures), and therefore, among the elective surgery, the roles of anemia in POD may not be as significant as in emergency surgery [40,41].

In this study, pre- and postoperative hemoglobin levels were almost in the normal range and there was no statistical difference between patients with POD (preoperative hemoglobin level: 12.7 g/dL, postoperative hemoglobin level: 11.5 g/dL) and patients without POD (preoperative hemoglobin level: 13.4 g/dL, postoperative hemoglobin level: 12 g/dL). Although anemia has been identified as a risk factor for delirium, the effect may be stronger in emergency surgery than in elective surgery [3].

Chou et al. have reported that patients with anemia on admission and who received a blood transfusion during operations were at higher risk of developing POD. However, patients without anemia on admission and who received a blood transfusion during operation were not. Therefore, there was no significant relationship between blood transfusions and POD in patients without anemia on admission [38]. In the same manner, blood transfusions did not have a statistically significant effect on the incidence of POD in this study. On the contrary, early restoration of unstable hemodynamics through blood transfusions could prevent POD. However, a further intervention study is needed to confirm the roles of blood transfusion in the development of POD.

This study has some limitations. In our hospital, POD was assessed by using the short form Korean Nursing Delirium Screening Scale on a daily basis by a nurse. Once patients were suspected to be POD by a nurse, the patients received psychiatric counseling and the diagnosis of POD was confirmed. Therefore, patients who had mild symptoms of POD and did not receive treatment by psychiatrists might have been excluded. Although standard delirium screening tools (Confusion Assessment Method or Delirium Rating Scale-R-98) were not used in our hospital, the diagnosis of POD had more accuracy due to the psychiatric consultation. This had an advantage in the analysis based on a solid diagnosis, but the low rate of diagnosis may have affected the analysis of the association among risk factors. In addition, patients who showed POD symptoms after discharge were not diagnosed and might have been excluded.

Another limitation was that data were retrospectively analyzed. Therefore, we could not examine brain lesions by brain imaging in all patients, and we could not analyze the severity of POD and cognitive dysfunction.

## 5. Conclusions

In this study, almost all risk factors including demographics, perioperative findings, laboratory findings, and medication history were evaluated using a CDW. The incidence of POD in spinal fusion surgery was 5.9%. There was no relationship between pre- and postoperative anemia and blood transfusion and the incidence of POD in spinal fusion surgery. Big data analytics using a CDW could be helpful for the comprehensive understanding of the risk factors of POD, and for preventing POD in spinal fusion surgery.

## Figures and Tables

**Table 1:** Preoperative, intraoperative and postoperative risk factors of patients.

	No Delirium (*n* = 3733)	Delirium (*n* = 234)	*p* Value
Preoperative factors			
Old Age (≥70 years), *n* (%)	1118 (28.9)	77 (73.3)	<0.001
Male, *n* (%)	1960 (50.8)	47 (44.8)	0.226
Body mass index, kg/m^2^ (median, IQR)	24.6 (22.5, 27)	24.5 (22.2, 26.8)	0.099
ASA physical status >2, *n* (%)	1065 (27.6)	64 (61.0)	<0.001
Emergency surgery (*n*, %)	296 (7.7)	4 (3.8)	0.14
Hypertension, *n* (%)	1720 (44.5)	59 (56.2)	0.018
Diabetes, *n* (%)	797 (20.6)	38 (36.2)	<0.001
Heart disease, *n* (%)	374 (9.7)	15 (14.3)	0.118
Stroke, *n* (%)	203 (5.3)	10 (9.5)	0.056
Cancer, *n* (%)	284 (7.4)	11 (10.5)	0.229
Parkinson disease, *n* (%)	29 (0.8)	2 (1.9)	0.185
Dementia, *n* (%)	27 (0.7)	4 (3.8)	<0.001
Depression, *n* (%)	78 (2.0)	6 (5.7)	0.009
Kidney disease, *n* (%)	137 (3.5)	4 (3.8)	0.886
Liver disease, *n* (%)	134 (3.5)	6 (5.7)	0.219
Insomnia, *n* (%)	163 (4.2)	9 (8.6)	0.031
Sleep disorder, *n* (%)	170 (4.4)	10 (9.5)	0.013
Alcohol, *n* (%)	1160 (30.0)	15 (14.3)	<0.001
Smoking, *n* (%)	773 (20.0)	14 (13.3)	0.09
Calcium channel blocker, *n* (%)	1507 (39.0)	44 (41.9)	0.55
Diuretics, *n* (%)	357 (9.2)	16 (15.2)	0.038
Beta blocker, *n* (%)	300 (7.8)	8 (7.6)	0.955
Angiotensin converting enzyme inhibitors, *n* (%)	18 (0.5)	0 (0.0)	0.483
Angiotensin receptor blockers, *n* (%)	205 (5.3)	4 (3.8)	0.498
Other anti-hypertension, *n* (%)	47 (1.2)	0 (0.0)	0.255
Miscellaneous drug, *n* (%)	269 (7.0)	7 (6.7)	0.906
Anti-depressant, *n* (%)	114 (3.0)	2 (1.9)	0.53
Hypnotics, *n* (%)	1350 (35.0)	39 (37.1)	0.643
Anti-psychotics, *n* (%)	131 (3.4)	17 (16.2)	<0.001
Non-steroidal anti-inflammatory drug, *n* (%)	2015 (54.0)	134 (57.3)	0.328
Analgesic except non-steroidal anti-inflammatory drug, *n* (%)	3508 (90.8)	102 (97.1)	0.026
Muscle relaxant, *n* (%)	1699 (44.0)	27 (25.7)	<0.001
Steroid, *n* (%)	1260 (32.6)	43 (41.0)	0.073
Anti-platelet, *n* (%)	21 (0.5)	0 (0.0)	0.449
Anti-coagulant, *n* (%)	2846 (73.7)	93 (88.6)	0.001
Anti-hyperlipidemic, *n* (%)	44 (1.1)	2 (1.9)	0.47
Anti-Parkinson, *n* (%)	6 (0.2)	0 (0.0)	0.686
Antihistamine, *n* (%)	1385 (35.9)	64 (61.0)	<0.001
Ant-vertigo, *n* (%)	2 (0.1)	0 (0.0)	0.816
Genitourinary drug, *n* (%)	2 (0.1)	0 (0.0)	0.816
H2 blocker, *n* (%)	1196 (31.0)	34 (32.4)	0.757
Preoperative hemoglobin, g/dL (median, IQR)	13.4 (12.4, 14.6)	12.7 (11.7, 13.8)	<0.001
Preoperative aspartate aminotransferase, units/L (median, IQR)	23 (19, 30)	23 (18, 32)	0.043
Preoperative alanine aminotransferase, units/L (median, IQR)	20 (15, 30)	19 (13, 28)	0.031
Preoperative sodium, mEq/L (median, IQR)	140 (138, 142)	140 (137, 141)	0.002
Preoperative potassium, mmol/L (median, IQR)	4.1 (3.9, 4.4)	4.2 (3.9, 4.5)	0.472
Preoperative uric acid, mg/dL (median, IQR)	4.7 (3.7, 5.8)	4.6 (3.7, 5.8)	0.706
Preoperative blood urea nitrogen, mg/dL (median, IQR)	15.6 (12.3, 19.3)	16.7 (12.8, 21.3)	0.018
Preoperative creatinine, mg/dL (median, IQR)	0.8 (0.7, 0.9)	0.8 (0.7, 1.0)	0.073
Preoperative albumin, g/dL (median, IQR)	4.3 (4, 4.5)	4 (3.7, 4.3)	<0.001
Intraoperative factors			
Anesthesia maintenance with propofol, *n* (%)	628 (16.3)	12 (11.4)	0.184
Surgical site reference: cervix, *n* (%)	1043 (27.0)	16 (15.2)	0.023
thorax, *n* (%)	297 (7.7)	11 (10.5)	
lumbar, *n* (%)	2522 (65.3)	78 (74.3)	
Surgical range, level (median, IQR)	2 (1, 2)	2(1,3)	<0.001
Operation time, hour (median, IQR)	3.4 (2.5, 4.4)	3.8 (2.8, 4.9)	<0.001
Intraoperative packed red blood cell, pack (median, IQR)	0 (0, 1)	1 (0, 2)	<0.001
Intraoperative fresh frozen plasma, pack (median, IQR)	0 (0, 0)	0 (0, 0)	0.012
Intraoperative platelet concentration, pack (median, IQR)	0 (0, 0)	0 (0, 0)	0.203
Opioid use, *n* (%)	3628 (93.9)	98 (93.3)	0.797
Midazolam, *n* (%)	111 (2.9)	6 (5.7)	0.09
Oliguria (<0.5 mL/kg/h), *n* (%)	381 (9.9)	7 (6.7)	0.276
Fluid balance (input–output), mL (median, IQR)	970 (550, 1500)	1170 (700, 1773.8)	<0.001
Estimated blood loss, mL (median, IQR)	500 (200, 800)	600 (500, 1000)	<0.001
Postoperative factors			
Postoperative packed red blood cell, pack (median, IQR)	0 (0, 0)	0 (0, 0)	0.003
Postoperative fresh frozen plasma, pack (median, IQR)	0 (0, 0)	0 (0, 0)	<0.001
Postoperative platelet concentration, pack (median, IQR)	0 (0, 0)	0 (0, 0)	0.026
Postoperative hemoglobin, g/dL (median, IQR)	12 (11, 13.2)	11.5 (10.6, 12.6)	<0.001
Postoperative aspartate aminotransferase, units/L (median, IQR)	26 (21, 32)	28 (22.8, 36.2)	0.013
Postoperative alanine aminotransferase, units/L (median, IQR)	18 (13, 26)	16 (12, 25)	0.368
Postoperative sodium, mEq/L (median, IQR)	140 (138, 142)	139 (137, 142)	0.001
Postoperative potassium, mmol/L (median, IQR)	3.9 (3.7, 4.2)	3.9 (3.7, 4.2)	0.517
Postoperative uric acid, mg/dL (median, IQR)	4 (3.1, 5.0)	3.9 (3.1, 4.9)	0.863
Postoperative blood urea nitrogen, mg/dL (median, IQR)	13 (10.6, 16.4)	14.6 (10.9, 18.2)	0.001
Postoperative creatinine, mg/dL (median, IQR)	0.7 (0.6, 0.9)	0.7 (0.6, 0.9)	0.212
Postoperative albumin, g/dL (median, IQR)	3.4 (3.1, 3.7)	3.2 (2.8, 3.4)	<0.001
Maximum body temperature, °C (median, IQR)	37.3 (37, 37.8)	37.4 (37.1, 37.8)	0.678
Postoperative erythrocyte sedimentation rate, mm/h (median, IQR)	14 (5, 31)	17 (5, 37)	0.063
Postoperative C-reactive protein, mg/L (median, IQR)	29.4 (3.8, 77.5)	50.3 (8.1, 103.3)	<0.001
Intensive-care unit, *n* (%)	649 (16.8)	39 (37.1)	<0.001
Patient-controlled analgesia, *n* (%)	3620 (93.7)	99 (94.3)	0.818

IQR, interquatile range.

**Table 2:** Intraoperative and postoperative red blood cell transfusion distributions.

Intraoperative RBC Transfusion, Pack			Postoperative RBC Transfusion, Pack		
	No Delirium, *n* (%)	Delirium, *n* (%)		No Delirium, *n* (%)	Delirium, *n* (%)
0	2701 (72.4)	115 (49.1)	0	3302 (88.5)	192 (82.1)
1	312 (8.4)	26 (11.1)	1	223 (6.0)	23 (9.8)
2	374 (10)	47 (20.1)	2	162 (4.3)	10 (4.3)
3	166 (4.4)	20 (8.5)	3	33 (0.9)	4 (1.7)
4	90 (2.4)	12 (5.1)	4	8 (0.2)	1 (0.4)
5	41 (1.1)	3 (1.3)	5≤	5 (0.1)	4 (1.7)
6	23 (0.6)	3 (1.3)			
7	7 (0.2)	3 (1.3)			
8	11 (0.3)	2 (0.9)			
9	2 (0.1)	0 (0)			
10≤	6 (0.2)	3 (1.3)			

RBC, red blood cell.

**Table 3:** The odds ratios of intraoperative and postoperative red blood cell transfusion (pack) for developing postoperative delirium.

	Delirium	
	Odds Ratio [95% Confidence Interval]	*p* Value
Intraoperative packed red blood cell, pack	1.10 (0.91–1.34)	0.313
Postoperative packed red blood cell, pack	0.91 (0.74–1.12)	0.379

**Table 4:** The odds ratios of preoperative, intraoperative and postoperative risk factors for developing postoperative delirium.

	Delirium	
	Odds Ratio (95% Confidence Interval)	*p* Value
Old Age (≥70 years)	3.08 (2.14 to 4.43)	<0.001
Male	1.77 (1.13 to 2.78)	0.013
Emergency surgery	1.19 (0.68 to 2.09)	0.531
ASA physical status >2	1.74 (1.21 to 2.49)	0.003
Anesthesia maintenance with propofol	1.09 (0.7 to 1.69)	0.718
Opioid use	0.68 (0.38 to 1.19)	0.176
Midazolam	2.12 (1.1 to 4.1)	0.026
Patient-controlled analgesia	1.19 (0.64 to 2.19)	0.581
Oliguria (<0.5 mL/kg/h)	0.81 (0.45 to 1.47)	0.494
Hypertension	1.09 (0.78 to 1.52)	0.606
Diabetes	1.08 (0.76 to 1.54)	0.667
Heart disease	0.73 (0.46 to 1.16)	0.181
Stroke	0.89 (0.51 to 1.54)	0.672
Cancer	0.52 (0.29 to 0.91)	0.023
Parkinson disease	5.54 (2.15 to 14.27)	<0.001
Dementia	4.5 (1.81 to 11.2)	0.001
Depression	2.8 (1.27 to 6.2)	0.011
Kidney disease	0.83 (0.37 to 1.85)	0.653
Liver disease	0.55 (0.21 to 1.43)	0.219
Insomnia	0.92 (0.37 to 2.25)	0.85
Sleep disorder	0.97 (0.42 to 2.24)	0.936
Alcohol	1.22 (0.89 to 1.67)	0.218
Smoking	1.01 (0.66 to 1.55)	0.958
Intensive care unit	3.45 (2.42 to 4.91)	<0.001
Calcium channel blocker	0.97 (0.71 to 1.32)	0.839
Diuretics	0.95 (0.59 to 1.53)	0.847
Beta blocker	0.9 (0.51 to 1.58)	0.706
Angiotensin converting enzyme inhibitors	0.67 (0.09 to 4.74)	0.685
Angiotensin receptor blockers	1.23 (0.65 to 2.35)	0.523
Other anti-hypertension	0.0 (0 to nan)	0.997
Miscellaneous drug	1.08 (0.6 to 1.94)	0.81
Anti-depressant	0.92 (0.36 to 2.37)	0.864
Hypnotics	0.93 (0.64 to 1.37)	0.728
Anti-psychotics	3.35 (1.91 to 5.89)	<0.001
Analgesic except non-steroidal anti-inflammatory drugs	0.87 (0.48 to 1.59)	0.661
Non-steroidal anti-inflammatory drug	1.11 (0.82 to 1.49)	0.496
Muscle relaxant	0.83 (0.59 to 1.16)	0.282
Steroid	1.14 (0.82 to 1.59)	0.431
Anti-platelet	1.08 (0.2 to 5.94)	0.927
Anti-coagulant	1.15 (0.77 to 1.72)	0.501
Anti-hyperlipidemic	1.38 (0.41 to 4.58)	0.6
Anti-Parkinson	0.0 (0 to nan)	0.999
Antihistamine	1.2 (0.82 to 1.76)	0.347
Ant-vertigo	0.0 (0 to nan)	0.999
Genitourinary drug	0.0 (0 to nan)	0.999
H2 blocker	1.13 (0.8 to 1.61)	0.48
Surgical site (reference: cervix)	nan (nan to nan)	0.298
Surgical site-thorax	1.35 (0.76 to 2.41)	0.306
Surgical site-lumbar	0.9 (0.59 to 1.38)	0.642
Intraoperative fresh frozen plasma, pack	0.83 (0.6 to 1.15)	0.262
Intraoperative platelet concentration, pack	0.95 (0.85 to 1.07)	0.385
Postoperative fresh frozen plasma, pack	1.21 (0.98 to 1.5)	0.074
Postoperative platelet concentration	1.02 (0.94 to 1.1)	0.672
Body mass index	1.0 (0.96 to 1.05)	0.854
Operation time	1.04 (0.92 to 1.18)	0.501
Preoperative hemoglobin	0.97 (0.83 to 1.14)	0.703
Postoperative hemoglobin	0.93 (0.78 to 1.11)	0.418
Preoperative aspartate aminotransferase	1.01 (1.0 to 1.02)	0.118
Postoperative aspartate aminotransferase	1.0 (0.99 to 1.01)	0.61
Preoperative alanine aminotransferase	0.99 (0.97 to 1.01)	0.26
Postoperative alanine aminotransferase	1.0 (0.98 to 1.02)	0.861
Preoperative sodium	1.0 (0.94 to 1.06)	0.914
Postoperative sodium	0.99 (0.94 to 1.05)	0.822
Preoperative potassium	1.19 (0.81 to 1.75)	0.372
Postoperative potassium	0.83 (0.58 to 1.18)	0.304
Preoperative uric acid	0.98 (0.83 to 1.15)	0.765
Postoperative uric acid	1.05 (0.87 to 1.26)	0.631
Preoperative blood urea nitrogen	0.98 (0.94 to 1.01)	0.151
Postoperative blood urea nitrogen	1.02 (0.98 to 1.06)	0.293
Preoperative creatinine	1.6 (0.84 to 3.07)	0.155
Postoperative creatinine	0.67 (0.34 to 1.3)	0.231
Preoperative albumin	0.52 (0.31 to 0.87)	0.012
Postoperative albumin	1.34 (0.78 to 2.33)	0.289
Maximum body temperature	1.04 (0.8 to 1.36)	0.779
Postoperative erythrocyte sedimentation rate	1.0 (0.99 to 1.01)	0.933
Postoperative C-reactive protein	1.0 (1.0 to 1.01)	0.078
Surgical range	0.94 (0.81 to 1.08)	0.381
Fluid balance (liter)	1.0 (1.0 to 1.0)	0.897
Estimated blood loss (liter)	1.0 (1.0 to 1.0)	0.905

## Data Availability

All data were obtained from the clinical data warehouse (CDW) of the five hospitals of Hallym University Medical Center.

## Appendix A. Predetermined Word List Describing Delirium

**Actually Investigated Words**	**Translation in English**
delirium	delirium
confusion	confusion
psychosis	psychosis
violent	violent
vigilant	vigilant
hyper alert	hyper alert
attention	attention
irritable	irritable
attentional deficit	attentional deficit
fluctuation	fluctuation
섬망	delirium
헛소리	gibberish, delirious talking
헛것	visual hallucination
지른다	yell
환시	visual hallucination
환청	auditory hallucination
환각	hallucination
망상	delusion
의식 변화	altered mentality
의식 혼탁	clouding of consciousness
집중력	concentration
외부 자극	external stimuli
외부 반응	response to a stimulus
반응성	response
이상행동	abnormal behavior
과다행동	hyperactive
행동변화	behavioral changes
공격	aggression
난폭	violence
충동적	impulsive
돌발행동	unexpected behavior
집중력	concentration
주의산만	distraction
횡설수설	gibberish
안절부절	restlessness
불안	restlessness
엉뚱	absurd
비논리적	irrational
상관없는	unconnected
각성	alertness
무기력	lethargy
인지능력	cognitive function
초조	restlessness
흥분	agitation
혼미	confusion
혼돈	confusion
혼란	confusion
지남력	orientation
불면	insomnia
요실금	urinary incontinence
졸림	drowsy
배회	wandering
혼잣말	self-talking
욕설	swear
고함	yell

## Appendix B. Drugs of Medication Factors

**Pharmacologic Category**	**Drug**
CCBs	Amlodipine, Barnidipine, Benidipine, Cilnidipine, Diltiazem, Efonidipine, Felodipine, Lacidipine, Lercanidipine Manidipine, Nicardipine, Nifedipine, Verapamil
Diuretics	Acetazolamide, Amiloride, Furosemide, hydrochlorothiazide, D-mannitol, Indapamide, Isosorbide, Spironolactone, Torasemide
Beta blockers	Arotinolol, Atenolol, Bisoprolol, Carvedilol, Labetalol, Metoprolol, Nebivolol, Propranolol
ACE inhibitors	Captopril, Cilazapril, Enalapril, Fosinopril Perindopril, Ramipril
ARBs	Candesartan, Eprosartan, Fimasartan Irbesartan, Losartan, Olmesartan, Telmisartan, Valsartan
Other antihypertensives	Amlodipine/Losartan, Ambrisentan, Bisoprolo/Hydrochlorothiazide, Bosentan. Candesartan/ Hydrochlorothiazide, Eprosartan/Hydrochlorothiazide, Felodipin/Ramipril, Fimasartan/Hydrochlorothiazide, Irbesartan/Hydrochlorothiazide, Lercanidipine/Valsartan, Losartan /Hydrochlorothiazide, Amlodipine/Olmesartan/Hydrochlorothiazide, Amlodipine/Olmesartan, Olmesartan/Hydrochlorothiazide, Sildenafil, Amlodipine/Telmisartan, Telmisartan/ Hydrochlorothiazide, Amlodipine/Valsartan, Valsartan/ Hydrochlorothiazide
Miscellaneous CV drugs	Amlodipine/Atorvastatin, Irbesartan/Atorvastatin, Ulinastatin, Ivabradine, Olmesartan/Rosuvastatin, Omega-3 acid ethyl esters, Telmisartan/Rosuvastatin, Trimetazidine, Pitavastatin/Valsartan
Antidepressants	Amitriptyline, Amoxapine, Doxepin, Imipramine, nortriptyline, Tianeptine, Fluvoxamine, Paroxetine, Escitalopram, Sertraline, Vortioxetine, Bupropion, Mirtazapine, Trazodone, Duloxetine, Desvenlafaxine, Milnacipran, Venlafaxine
Hypnotics and sedatives	Alprazolam, Buspirone, Chlordiazepoxide, Chlordiazepoxide/Clidinium, Clobazam Chloral, Diazepam, Etizolam, Flurazepam, Dexmedetomidine, Lorazepam, Midazolam, Phenobarbital, Melatonin, Tandospirone, Triazolam, Zolpidem
Antipsychotics	Amisulpride, Aripiprazole, Blonanserin, Clozapine, Chlorpromazine, Haloperidol, Paliperidone, Risperidone, Olanzapine, Paliperidone, Quetiapine, Sulpiride, Ziprasidone
CNS stimulants	Atomoxetine, Clonidine, Modafinil, Methylphenidate
Antimanic agents	Lithium
Opioids	Buprenorphine, Fentanyl, Morphine, Oxycodone, Pethidine Sufentanil, Hydromorphone, Oxycodone,
Other analgesics (no NSAIDs)	Acetaminophen, Capsaicin, Ethanol, Nefopam, Propacetamol, Tramadol
NSAIDs	Aceclofenac, Celecoxib, Dexibuprofen, Diclofenac, Etoricoxib, Ibuprofen, Imidazole salicylate, Indomethacin, Ketoprofen, Ketorolac, Loxoprofen, Meloxicam, Nabumetone, Naproxen, Pelubiprofen, Polmacoxib, Zaltoprofen
Skeletal muscle relaxants	Afloqualone, Baclofen, Cyclobenzaprine, Dantrolene, Eperisone, Clostridium botulinum A toxin, Cisatracurium, Gallamine, Rocuronium, Suxamethonium, Vecuronium, Orphenadrine, Chlorphenesin, Thiocolchicoside, Aescin, Tizanidine
Corticosteroids	Budesonide, Deflazacort, Dexamethasone, Fludrocortisone, Hydrocortisone, Methylprednisolone, Triamcinolon, Prednisolone
Antiplatelet agents	Aspirin, Cilostazol, Cilostazol/Ginkgo Biloba Leaf Extract, Clopidogrel, Clopidogrel/Aspirin, Abciximab, Prasugrel, Sulodexide, Ticlopidine, Ticagrelor, Triflusal
Anticoagulants	Human Antithrombin Ⅲ, Enoxaparin, Gabexate, Heparin, Tirofiban, Warfarin, Tinzaparin, Apixaban, Dabigatran, Edoxaban, Argatroban, Rivaroxaban
Antihyperlipidemic agents	Atorvastatin/Ezetimibe, Atorvastatin, Cholestyramine, Ezetimibe, Fenofibric acid, Fluvastatin, Gemfibrozil, Lovastatin, Pitavastatin, Pravastatin/Fenofibrate, Rosuvastatin, Rosuvastatin/Ezetimibe, Simvastatin/ Ezetimibe, Simvastatin/Fenofibrate, Simvastatin
Antiparkinsonian agents	Amantadine, Levodopa/Benserazide, Bromocryptine, Benztropine, Levodopa/Carbidopa/Entacapone, Levodopa/Carbidopa, Entacapone, Pramipexole, Procyclidine, Rasagiline, Ropinirole, Selegiline, Trihexyphenidyl
Antihistamines/antiallergics	Azelastine, Levocetirizine, Chlorpheniramin, Dimenhydrinate, Desloratadine, Ebastine, Pseudoephedrine, Emedastine, Epinastine, Fexofenadine, γ-Linolenic acid, Hydroxyzine, Chlorpheniramine, Human IgG/Histamine, Ketotifen, Purified House Dust Mite allergen extract, Loratadine, Mequitazine, Olopatadine, Allergen extracts, Tranilast
Antivertigos	Betahistine, Cinnarizine/Dimenhydrinate
Genitourinary Smooth Muscle Relaxants	Fesoterodine, Atosiban, Mirabegron, Oxybutynin, Propiverine, Solifenacin, Trospium
H2 receptor antagonist	Cimetidine, Famotidine, Lafutidine, Nizatidine, Ranitidine
CCBs, calcium channel blockers; ACE inhibitors: agiotensin-converting enzyme inhibitors; ARBs, angiotension II receptor blockers; CV, cardiovascular; NSAIDs, non-steroidal anti-inflammatory drugs.	
